# ChatGPT as a Source of Information for Bariatric Surgery Patients: a Comparative Analysis of Accuracy and Comprehensiveness Between GPT-4 and GPT-3.5

**DOI:** 10.1007/s11695-024-07212-6

**Published:** 2024-04-02

**Authors:** Jamil S. Samaan, Nithya Rajeev, Wee Han Ng, Nitin Srinivasan, Jonathan A. Busam, Yee Hui Yeo, Kamran Samakar

**Affiliations:** 1 Karsh Division of Digestive and Liver Diseases, Department of Medicine, Cedars-Sinai Medical Center, 8700 Beverly Blvd, Los Angeles, CA 90048 USA; 2 Division of Upper GI and General Surgery, Department of Surgery, Keck School of Medicine of USC, Health Care Consultation Center, 1510 San Pablo St #514, Los Angeles, CA 90033 USA; 3 Bristol Medical School, University of Bristol, 5 Tyndall Ave, Bristol, BS8 1UD UK

**Keywords:** ChatGPT, GPT-4, GPT-3.5, Surgery, Bariatric surgery, Weight loss, Artificial intelligence

## Introduction

Bariatric surgery is an effective and safe treatment for severe obesity [1]. Accurate and comprehensive perioperative education is integral to patients’ surgical journeys and outcomes. Large language models (LLMs), like ChatGPT, have the potential to revolutionize patient education by leveraging vast quantities of data to respond to user prompts in an easy-to-understand and conversational manner. Released by OpenAI in November of 2022, GPT-3.5 acquired 1 million users within 5 days of its release, outpacing applications such as Facebook, Twitter, and Instagram [2]. By January of 2023, its user base reached 100 million monthly active users, making it the fastest growing consumer application in history [3]. Our recent study demonstrated the impressive ability of GPT-3.5 in answering questions related to bariatric surgery, showing high accuracy, comprehensiveness, and reproducibility of responses [4]. GPT-3.5’s successor, GPT-4, was released in March of 2023 with improvements in performance across multiple domains [5–8]. The current study builds on our previous analysis by comparing the accuracy and comprehensiveness of GPT-4 compared to GPT-3.5, in answering questions related to bariatric surgery.

## Methods

A total of 151 questions related to bariatric surgery sourced from healthcare institutions, and Facebook support groups were included. The methodology for question curation is described in our previous study [4]. To better characterize ChatGPT’s performance, questions were organized into 5 categories: (1) “eligibility, efficacy, and procedure options”, (2) “preoperative preparation”, (3) “recovery, risks, and complications”, (4) “lifestyle changes”, and (5) “other”.

### Response Generation and Grading

Each question was entered independently into both GPT-3.5 and GPT-4 in July 2023 using the “New Chat” function on the OpenAI platform. Differences in accuracy and comprehensiveness of responses between GPT-3.5 and GPT-4 were graded by a board-certified, fellowship-trained, bariatric surgeon practicing in a tertiary and quaternary referral center with over 10 years of experience. The scale used for independent grading of accuracy and comprehensiveness was as follows: Compared to the response from GPT-3.5, the response from GPT-4 is:

1. Less accurate/comprehensiveness
2. Similar accuracy/ comprehensiveness
3. More accurate/comprehensive

Statistical analysis consisted of descriptive analysis summarizing proportions and percentages of responses earning each grade. All statistical analyses were performed in Microsoft Excel (Version 16.69.1).

## Results

**Fig. 1:**
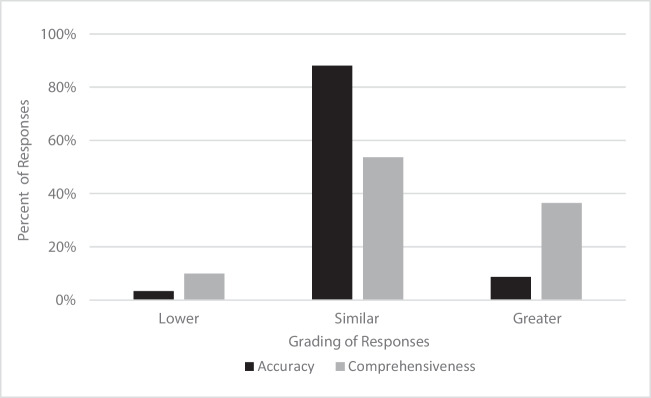
Accuracy and comprehensiveness of responses generated by GPT-4.0 compared to GPT-3.5 to questions related to bariatric surgery

A total of 151 questions were included in our analysis (Supplementary Table 1). The majority of responses were graded as similar in accuracy between the two models. Of the total 151 responses from GPT-4, 3 (3.3%) were graded as less accurate, 133 (88.1%) as similar in accuracy, and 13 (8.6%) as more accurate compared to GPT-3.5 (Table 1, Fig. 1). A more notable difference in responses was observed when examining the comprehensiveness between the two models. A total of 15/151 (9.9%) of GPT-4’s responses were graded as less comprehensive, 81/151 (53.6%) as similar comprehensiveness, and 55/151 (36.4%) as more comprehensive compared to GPT-3.5 (Table 1, Fig. 1).

**Table 1:** Accuracy and comprehensiveness of responses generated by GPT-4.0 compared to GPT-3.5 to questions related to bariatric surgery stratified by question category

	Accuracy of GPT-4.0 vs GPT-3.5			Comprehensiveness of GPT-4.0 vs GPT-3.5		
	Number of responses (%)			Number of responses (%)		
Question category	Lower	Similar	Greater	Lower	Similar	Greater
Eligibility, efficacy and procedure options (*N* = 32)	0 (0)	30 (93.8)	2 (6.3)	3 (9.4)	14 (43.8)	15 (46.9)
Preoperative preparation (*N* = 15)	2 (13.3)	11 (73.3)	2 (13.3)	3 (20)	5 (33.3)	7 (46.7)
Recovery, risks and complications (*N* = 75)	3 (4)	64 (85.3)	8 (10.7)	6 (8)	44 (58.7)	25 (33.3)
Lifestyle Changes (*N* = 17)	0 (0)	16 (94.1)	1 (5.9)	2 (11.8)	11 (64.7)	4 (23.5)
Others (*N* = 12)	0 (0)	12 (100)	0 (0)	1 (8.3)	7 (58.3)	4 (33.3)
Total (*N* = 151)	5 (3.3)	133 (88.1)	13 (8.61)	15 (9.9)	81 (53.6)	55 (36.4)

## Conclusion

We present a follow up analysis comparing the accuracy and comprehensiveness of responses from GPT-3.5 and GPT-4 to questions related to bariatric surgery. In terms of accuracy, our results show a largely uniform performance between the two models with a significant majority (88.1%) of responses graded as having similar accuracy. These findings may suggest a degree of stability and reliability among the core algorithms when it comes to the generation of accurate responses. It is important to note that both models have been undergoing continuous refinement and updating, which may explain this comparability in performance. A more striking differentiation was observed when examining the comprehensiveness of responses. While over half of the responses (53.6%) had similar levels of comprehensiveness between the two models, a considerable number (36.4%) of GPT-4’s responses were found to be more comprehensive compared to GPT-3.5. This could be attributed to the enhanced training methodologies and an expanded data set in GPT-4, allowing for more context-rich and detailed answers [5]. For example, in "Preoperative Preparation," GPT-4 provided an extensive list of pre-surgical dietary guidelines as well as psychosocial considerations that were absent in GPT-3.5’s response. It’s notable that GPT-4 provided less comprehensive and accurate responses compared to GPT-3.5 for some questions. This discrepancy in performance for a minority of questions may be due to multiple reasons including model training, training data, and the nature of LLMs which generate text based on probabilities, leading to variation in performance on some occasions. Our study design was pragmatic in that question input mirrored how a user with no technological background may use an LLM. Therefore, advanced prompting strategies may minimize the variation in performance of LLMs and improve overall performance, a topic that would benefit from investigation in future studies.

### Limitations and Future Directions

Our study is not without its limitations. First, the grading of responses was carried out by a single reviewer, which is subjective in nature despite the reviewer’s extensive experience. The list of questions used in our study is not comprehensive of all possible patient questions related to bariatric surgery and therefore may not be generalizable to ChatGPTs responses to all possible information regarding bariatric surgery.

In conclusion, GPT-3.5 and GPT-4 demonstrated relatively similar ability to generate accurate responses to bariatric surgery-related questions. However, GPT-4 provided more comprehensive responses to 36.4% of questions, demonstrating a significant improvement in model performance with iterations of the ChatGPT model. It’s important to note that both models provided inaccurate information, and therefore we advocate for their potential future role as adjunct sources of information to medical advice provided by licensed healthcare professionals. Our analysis suggests a steady increase in the robustness of large language models in providing accurate and comprehensive medical information. These improvements may be significant in future iterations and warrant further studies to examine their impact on clinical outcomes in bariatric surgery.

### Supplementary Information

Below is the link to the electronic supplementary material.

Supplementary file1 (DOCX 221 KB)
